# Supplementary material for: Population structure and genetic diversity of Tamarix chinensis as revealed with microsatellite markers in two estuarine flats
Source: PeerJ. 2023 Sep 11;11:e15882. doi: 10.7717/peerj.15882 (PMC10501381; doi:10.7717/peerj.15882)
Supplement: Supplemental Information 1 [file peerj-11-15882-s001.docx]

| Primer | Sense（5′-3′） | Anti-Sense (5′-3′） | Tm (℃) | Repeat | Amplicon size |
| --- | --- | --- | --- | --- | --- |
| Essr1 | GGGTGGACGACTTGGC | CGTGCGTGCGTATCAA | 49.8 | (ATT)_6_ | 170 |
| Essr2 | AACCACCGATACATACAGA | TGACAAATGACCTCCAAG | 49.6 | (ATCT)_5_ | 270 |
| Essr3 | GCGGTGCTTCCTTCATCT | CAAATGGGAGTGATGATGG | 58.5 | (TCC)_6_ | 205 |
| Essr4 | GGGCAATGTTTCCGATTC | AGGCGACTGTAGCCAAGC | 49.8 | (AGCAGT)_4_ | 190 |
| Essr5 | CATTTAGGACGAAACGAA | CACGAGTGGCTTGACAGA | 53.2 | (TA)_9_ | 187 |
| Essr6 | GTCCGTCGCTCTTGTATTT | GGTGATTGAAGCCATTGTTAT | 58.3 | (TC)_9_ | 189 |
| Gssr1/tc3 | AAAGCAGGTGAGATTGAA | ACACCCTAATCCACATAAC | 55 | (TTA)_11_ | 177 |
| Gssr2/tc5 | GTCTGCCTAAGAAGTCGC | CGGAAATAAGGGAGAAAT | 55 | (TCTT)_8_ | 173 |
| Gssr3 | AATTCAAAGTTTTTCCGTTCCTC | TATCGCTCTCACTTTCCCTAACA | 52 | (AAAT)_5_ | 146 |
| Gssr4 | ATGAGAAATGATGGTAAGCTGGA | TGTGGCTTTGAAGGTTGACTTAT | 52 | (TTTA)_5_ | 129 |
| Gssr5 | AGATGGTAATTGTGTTTATGCGG | GCCAAACAAGGGGTTAATTTTT | 55 | (ATAG)_7_ | 171 |
| Gssr6/tc8 | TTTGAGTTTGACGATGTA | GATTGACCGTGTTTTAGT | 52 | (AAT)_11_ | 214 |
