# Supplementary material for: Population structure and genetic diversity of Tamarix chinensis as revealed with microsatellite markers in two estuarine flats
Source: PeerJ. 2023 Sep 11;11:e15882. doi: 10.7717/peerj.15882 (PMC10501381; doi:10.7717/peerj.15882)
Supplement: Supplemental Information 2 — yes, the presence of null alleles; no, the absence of null alleles [file peerj-11-15882-s002.docx]

|  | YHK | CY | FS | YDG | YXX | YHD | HHJ | HLS | HCX |
| --- | --- | --- | --- | --- | --- | --- | --- | --- | --- |
| Essr1 | no | no | no | no | no | no | no | no | no |
| Essr2 | no | no | yes | no | no | no | no | no | yes |
| Essr3 | yes | no | no | no | no | no | no | no | no |
| Essr4 | yes | no | no | no | no | no | no | no | yes |
| Essr5 | yes | no | no | no | no | yes | no | no | no |
| Essr6 | no | no | no | no | no | no | no | no | no |
| Gssr1 | no | no | no | no | no | no | no | no | no |
| Gssr2 | no | no | no | no | no | no | yes | no | no |
| Gssr3 | yes | no | no | no | no | yes | no | no | yes |
| Gssr4 | no | no | no | no | no | yes | no | no | no |
| Gssr5 | yes | no | no | no | no | yes | no | no | no |
| Gssr6 | no | no | no | no | no | no | no | no | no |
