# Supplementary material for: Population structure and genetic diversity of Tamarix chinensis as revealed with microsatellite markers in two estuarine flats
Source: PeerJ. 2023 Sep 11;11:e15882. doi: 10.7717/peerj.15882 (PMC10501381; doi:10.7717/peerj.15882)
Supplement: Supplemental Information 3 — ∗P < 0.05, ∗∗P < 0.01 [file peerj-11-15882-s003.docx]

|  | YHK | CY | FS | YDG | YXX | YHD | HHJ | HLS | HCX |
| --- | --- | --- | --- | --- | --- | --- | --- | --- | --- |
| Essr1 | 0.017 | 0.144 | -0.245 | -0.003 | 0.160 | -0.134 | -0.133 | 0.128 | -0.223^*^ |
| Essr2 | 0.148 | 0.185 | 0.375 | -0.125^*^ | -0.331 | 0.020 | -0.310 | 0.612^**^ | 0.165 |
| Essr3 | 0.005 | 0.033 | -0.055 | -0.305^*^ | -0.312^*^ | -0.358 | -0.230 | -0.289 | -0.052 |
| Essr4 | 0.490^**^ | -0.057 | -0.333 | -0.408 | 0.098 | 0.210 | -0.148 | 0.453^**^ | 0.581^**^ |
| Essr5 | 0.321^*^ | -0.112 | 0.119 | -0.134 | 0.318^**^ | 0.059 | 0.124 | 0.233 | 0.073 |
| Essr6 | 0.149^*^ | -0.156 | -0.104 | 0.151^*^ | -0.111 | -0.083 | -0.089^**^ | 0.057 | 0.117^**^ |
| Gssr1 | 0.047 | -0.023 | -0.049 | 0.117 | 0.043 | 0.237^**^ | -0.095 | -0.04^5*^ | -0.222 |
| Gssr2 | 0.239^**^ | 0.159^*^ | 0.122^*^ | 0.128^**^ | 0.044 | 0.192 | 0.089^*^ | 0.037^*^ | 0.046 |
| Gssr3 | 0.056 | -0.024 | -0.056 | 0.095 | 0.057 | 0.218^**^ | -0.084 | -0.02^5*^ | -0.276 |
| Gssr4 | 0.054 | 0.163^*^ | 0.117* | 0.135^**^ | 0.041 | 0.159 | 0.074^*^ | 0.041^*^ | 0.043 |
| Gssr5 | 0.043 | -0.046 | -0.067 | 0.121 | 0.053 | 0.196^**^ | -0.097 | -0.03^5*^ | -0.162 |
| Gssr6 | 0.169^*^ | 0.139^*^ | 0.117* | 0.123^**^ | 0.049 | 0.173 | 0.070^*^ | 0.041^*^ | 0.055 |
