# Supplementary material for: Population structure and genetic diversity of Tamarix chinensis as revealed with microsatellite markers in two estuarine flats
Source: PeerJ. 2023 Sep 11;11:e15882. doi: 10.7717/peerj.15882 (PMC10501381; doi:10.7717/peerj.15882)
Supplement: Supplemental Information 4 [file peerj-11-15882-s004.docx]

|  | YHK | CY | FS | YDG | YXX | YHD | HHJ | HLS | HCX |
| --- | --- | --- | --- | --- | --- | --- | --- | --- | --- |
| Chi2 | 50.6677 | 21.1335 | 26.6171 | 43.7849 | >49.1328 | 28.9144 | 35.9347 | >70.8260 | >59.3780 |
| Df | 16 | 16 | 16 | 16 | 16 | 16 | 16 | 16 | 16 |
| Prob | 1.79E-05 | 0.17343 | 0.04594 | 0.000213 | <3.15e-05 | 0.02452 | 0.002955 | <7.15e-09 | <6.66e-0 |
