# Supplementary material for: Population structure and genetic diversity of Tamarix chinensis as revealed with microsatellite markers in two estuarine flats
Source: PeerJ. 2023 Sep 11;11:e15882. doi: 10.7717/peerj.15882 (PMC10501381; doi:10.7717/peerj.15882)
Supplement: Supplemental Information 5 [file peerj-11-15882-s005.docx]

| Pop | Locus#1 | Locus#2 | P-Value | S.E. | Switches |
| --- | --- | --- | --- | --- | --- |
| YHK | Essr3 | Essr4 | 0.01103 | 0.001725 | 24409 |
| CY | Essr1 | Essr5 | 0 | 0 | 1488 |
| CY | Essr3 | Gssr3 | 0.01877 | 0.004776 | 6123 |
| CY | Essr4 | Gssr5 | 0.00547 | 0.003708 | 3643 |
| CY | Gssr1 | Gssr2 | 0.00005 | 0.00005 | 1455 |
| FS | Essr5 | Essr6 | 0.00842 | 0.00664 | 872 |
| FS | Essr5 | Gssr1 | 0 | 0 | 1700 |
| FS | Essr1 | Gssr2 | 0.02221 | 0.007439 | 2664 |
| FS | Essr5 | Gssr6 | 0.0048 | 0.002936 | 1699 |
| HHJ | Essr3 | Essr4 | 0.0095 | 0.004484 | 4802 |
| HHJ | Essr1 | Essr5 | 0 | 0 | 1427 |
| HHJ | Essr2 | Essr5 | 0.01744 | 0.008411 | 2875 |
| HHJ | Essr1 | Essr6 | 0 | 0 | 1656 |
| HHJ | Essr2 | Essr6 | 0 | 0 | 3341 |
| HHJ | Essr3 | Essr6 | 0.00218 | 0.001089 | 3578 |
| HHJ | Essr4 | Essr6 | 0.02014 | 0.00756 | 4386 |
| HHJ | Essr5 | Essr6 | 0 | 0 | 1726 |
| HHJ | Essr1 | Gssr1 | 0.00687 | 0.004151 | 3641 |
| HHJ | Essr2 | Gssr1 | 0.00672 | 0.002239 | 7339 |
| HHJ | Essr3 | Gssr1 | 0.01029 | 0.004253 | 6377 |
| HHJ | Essr4 | Gssr3 | 0.00209 | 0.001079 | 6300 |
| HHJ | Essr5 | Gssr5 | 0 | 0 | 4325 |
| HHJ | Essr6 | Gssr5 | 0 | 0 | 5293 |
| HHJ | Essr1 | Gssr2 | 0.00319 | 0.002985 | 4054 |
| HHJ | Essr6 | Gssr2 | 0.00062 | 0.00062 | 4448 |
| HLS | Essr1 | Gssr2 | 0.00204 | 0.001184 | 2989 |
| HLS | Essr5 | Gssr4 | 0.01747 | 0.004064 | 6048 |
| HLS | Essr6 | Gssr6 | 0.02255 | 0.009887 | 2364 |
| HCX | Essr2 | Essr5 | 0.01079 | 0.003637 | 3638 |
| HCX | Essr5 | Gssr6 | 0.00354 | 0.002687 | 1586 |
