# Supplementary material for: Population structure and genetic diversity of Tamarix chinensis as revealed with microsatellite markers in two estuarine flats
Source: PeerJ. 2023 Sep 11;11:e15882. doi: 10.7717/peerj.15882 (PMC10501381; doi:10.7717/peerj.15882)
Supplement: Supplemental Information 6 [file peerj-11-15882-s006.docx]

| Locus#1 | Locus#2 | chi2 | df | P-Value |
| --- | --- | --- | --- | --- |
| Essr3 | Essr4 | 32.847190 | 18 | 0.017415 |
| Essr1 | Essr5 | >73.024925 | 18 | <1.38e-08 |
| Essr1 | Essr6 | >49.208969 | 18 | <9.93e-05 |
| Essr2 | Essr6 | >54.022716 | 18 | <1.82e-05 |
| Essr5 | Essr6 | >60.037371 | 18 | <2.02e-06 |
| Essr3 | Gssr1 | 34.170150 | 18 | 0.012 |
| Essr5 | Gssr1 | >84.304844 | 18 | <1.50e-10 |
| Essr6 | Gssr1 | >60.721954 | 18 | <1.56e-06 |
| Essr1 | Gssr2 | 44.333878 | 18 | 0.000517 |
| Essr5 | Gssr2 | 45.183495 | 18 | 0.00039 |
| Essr6 | Gssr2 | 31.900703 | 18 | 0.02259 |
| Gssr1 | Gssr2 | 54.062159 | 18 | 1.79E-05 |
