# Supplementary material for: Population structure and genetic diversity of Tamarix chinensis as revealed with microsatellite markers in two estuarine flats
Source: PeerJ. 2023 Sep 11;11:e15882. doi: 10.7717/peerj.15882 (PMC10501381; doi:10.7717/peerj.15882)
Supplement: Supplemental Information 7 [file peerj-11-15882-s007.docx]

|  | YHK | CY | FS | YDG | YXX | YHD | HHJ | HLS | HCX |
| --- | --- | --- | --- | --- | --- | --- | --- | --- | --- |
| YHK | 0.000 |  |  |  |  |  |  |  |  |
| CY | 0.039 | 0.000 |  |  |  |  |  |  |  |
| FS | 0.029 | 0.022 | 0.000 |  |  |  |  |  |  |
| YDG | 0.021 | 0.024 | 0.025 | 0.000 |  |  |  |  |  |
| YXX | 0.022 | 0.020 | 0.027 | 0.017 | 0.000 |  |  |  |  |
| YHD | 0.017 | 0.019 | 0.021 | 0.030 | 0.014 | 0.000 |  |  |  |
| HHJ | 0.041 | 0.047 | 0.033 | 0.034 | 0.030 | 0.030 | 0.000 |  |  |
| HLS | 0.046 | 0.059 | 0.054 | 0.044 | 0.046 | 0.049 | 0.037 | 0.000 |  |
| HCX | 0.061 | 0.110 | 0.060 | 0.055 | 0.050 | 0.054 | 0.046 | 0.039 | 0.000 |
