# Supplementary material for: Population structure and genetic diversity of Tamarix chinensis as revealed with microsatellite markers in two estuarine flats
Source: PeerJ. 2023 Sep 11;11:e15882. doi: 10.7717/peerj.15882 (PMC10501381; doi:10.7717/peerj.15882)
Supplement: Supplemental Information 8 [file peerj-11-15882-s008.docx]

| **K** | **Reps** | **Mean LnP(K)** | **Stdev LnP(K)** | **Ln'(K)** | **\|Ln''(K)\|** | **Delta K** |
| --- | --- | --- | --- | --- | --- | --- |
| 1 | 10 | -541.760000 | 0.374759 | — | — | — |
| 2 | 10 | -275.130000 | 2.378165 | 266.630000 | 172.190000 | 72.404577 |
| 3 | 10 | -180.690000 | 1.292242 | 94.440000 | 67.630000 | 52.335406 |
| 4 | 10 | -153.880000 | 25.659947 | 26.810000 | 154.210000 | 6.009755 |
| 5 | 10 | -281.280000 | 85.135380 | -27.400000 | 256.380000 | 3.011439 |
| 6 | 10 | -152.300000 | 8.575935 | 128.980000 | 265.340000 | 30.940065 |
| 7 | 10 | -288.660000 | 95.747401 | -36.360000 | 30.310000 | 0.316562 |
| 8 | 10 | -455.330000 | 149.375731 | -66.670000 | 131.520000 | 0.880464 |
| 9 | 10 | -490.480000 | 160.259497 | -35.150000 | 144.200000 | 0.899791 |
| 10 | 10 | -669.830000 | 325.799605 | -79.350000 | — | — |
