# Supplementary material for: Population structure and genetic diversity of Tamarix chinensis as revealed with microsatellite markers in two estuarine flats
Source: PeerJ. 2023 Sep 11;11:e15882. doi: 10.7717/peerj.15882 (PMC10501381; doi:10.7717/peerj.15882)
Supplement: Supplemental Information 9 [file peerj-11-15882-s009.docx]

| population | Wilcoxon’s sign-rank test | | Mode-shift test (distribution shape) |
| --- | --- | --- | --- |
|  | TMP | SMM |  |
| **YHK** | 0.3203 | 0.5781 | L-shaped |
| **CY** | 0.2305 | 0.7695 | L-shaped |
| **FS** | 0.8438 | 0.9902 | L-shaped |
| **YDG** | 0.2734 | 0.8750 | L-shaped |
| **YXX** | 0.2305 | 0.8086 | L-shaped |
| **YHD** | 0.7265 | 0.9805 | L-shaped |
| **HHJ** | 0.8086 | 0.9914 | L-shaped |
| **HLS** | 0.3203 | 0.8750 | L-shaped |
| **HCX** | 0.1250 | 0.6289 | L-shaped |
