# Supplementary material for: Population structure and genetic diversity of Tamarix chinensis as revealed with microsatellite markers in two estuarine flats
Source: PeerJ. 2023 Sep 11;11:e15882. doi: 10.7717/peerj.15882 (PMC10501381; doi:10.7717/peerj.15882)
Supplement: Supplemental Information 10 [file peerj-11-15882-s010.docx]

| **Pop** | **N** | **Na** | **Ne** | **Ar** | **Np** | **I** | **Ho** | **He** | **uHe** | **Fis** |
| --- | --- | --- | --- | --- | --- | --- | --- | --- | --- | --- |
| **YHK** | 20.875 | 8.000 | 4.984 | 9.25 | 2 | 1.651 | 0.570 | 0.690 | 0.709 | 0.177 |
| **YDG** | 22.500 | 8.375 | 5.325 | 9.84 | 3 | 1.727 | 0.746 | 0.712 | 0.730 | -0.072 |
| **YXX** | 22.250 | 9.500 | 5.914 | 10.38 | 7 | 1.854 | 0.743 | 0.739 | 0.758 | -0.011 |
| **YHD** | 21.125 | 8.375 | 4.953 | 9.85 | 7 | 1.693 | 0.687 | 0.700 | 0.718 | 0.018 |
| **HHJ** | 21.375 | 7.250 | 3.978 | 8.56 | 5 | 1.547 | 0.749 | 0.678 | 0.695 | -0.099 |
| **HLS** | 17.000 | 7.750 | 4.560 | 8.76 | 6 | 1.648 | 0.586 | 0.702 | 0.725 | 0.148 |
| **HCX** | 25.250 | 8.375 | 5.047 | 9.27 | 16 | 1.698 | 0.662 | 0.706 | 0.721 | 0.061 |
| **AverY^*^** | 21.687 | 8.563 | 5.294 | 9.83 | 4.75 | 1.731 | 0.680 | 0.710 | 0.729 | 0.028 |
| **AverH^*^** | 21.208 | 7.791 | 4.528 | 8.86 | 9 | 1.631 | 0.666 | 0.695 | 0.714 | 0.037 |
