# Supplementary figures and images for: Population structure and genetic diversity of Tamarix chinensis as revealed with microsatellite markers in two estuarine flats
Source: PeerJ. 2023 Sep 11;11:e15882. doi: 10.7717/peerj.15882 (PMC10501381; doi:10.7717/peerj.15882)

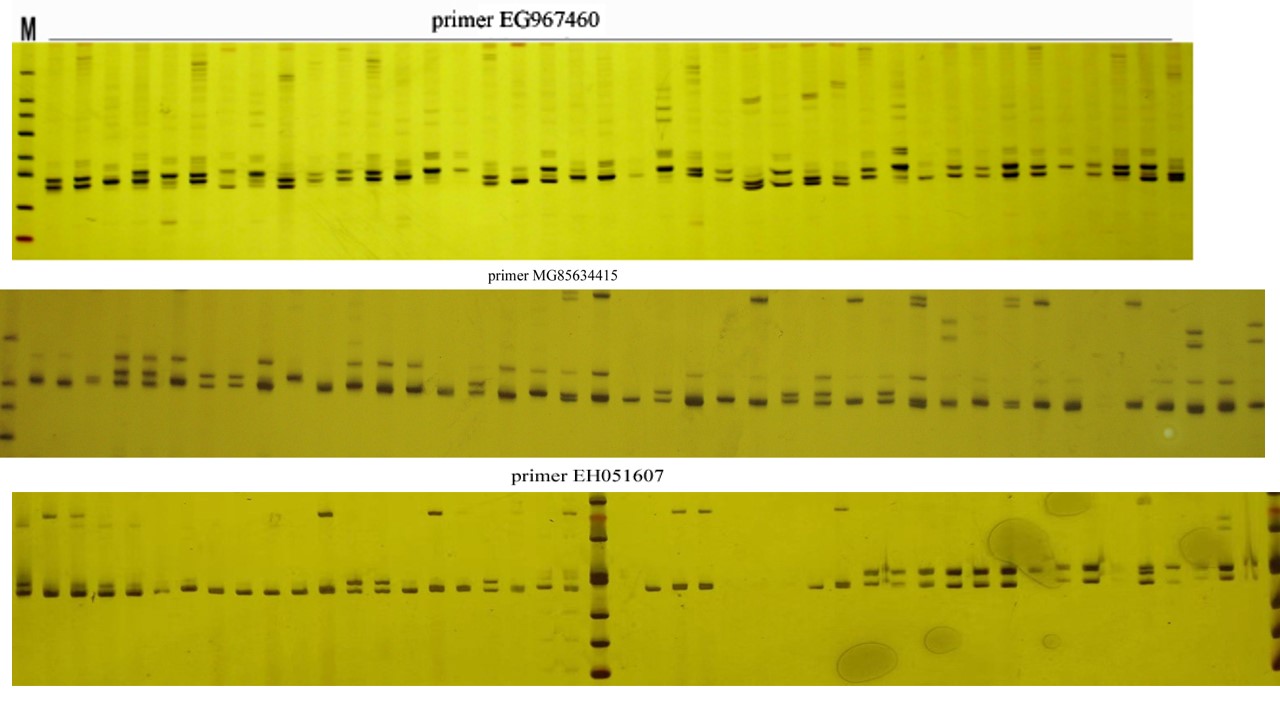

Supplement: Supplemental Information 12 [file peerj-11-15882-s012.jpg]
