# Supplementary material for: Population structure and genetic diversity of Tamarix chinensis as revealed with microsatellite markers in two estuarine flats
Source: PeerJ. 2023 Sep 11;11:e15882. doi: 10.7717/peerj.15882 (PMC10501381; doi:10.7717/peerj.15882)
Supplement: Supplemental Information 13 [file peerj-11-15882-s013.docx]

**Job billowing-tree-605c**

This output file was generated at:

2022-Feb-09 17:22:27 PST

**This document is not permanent.** It will automatically be removed from the server in seven (7) days. Please save or print it for your records. If images are missing, try reloading; this sometimes happens under heavy server load.

Single file archive including this page, all images, all clumpp files: [download](http://taylor0.biology.ucla.edu/structureHarvester/completedJobs/billowing-tree-605c/archive.tar.gz). [.tar.gz]

**L(K)**


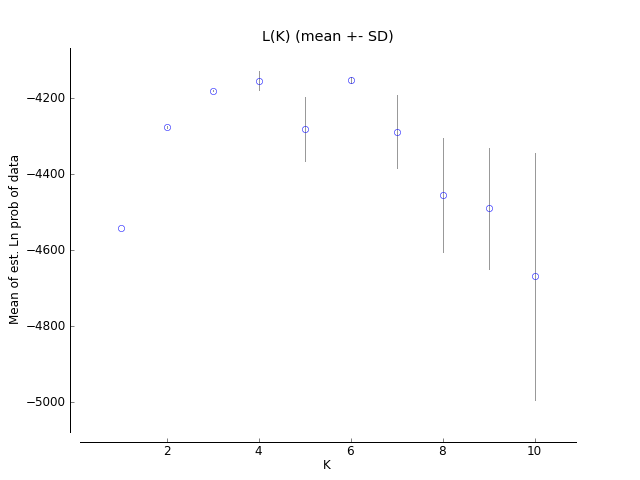


L(K): [pdf](http://taylor0.biology.ucla.edu/structureHarvester/completedJobs/billowing-tree-605c/meanLnProb.pdf)   [eps](http://taylor0.biology.ucla.edu/structureHarvester/completedJobs/billowing-tree-605c/meanLnProb.eps)

**Clumpp files**

[K = 1 Clumpp indfile](http://taylor0.biology.ucla.edu/structureHarvester/completedJobs/billowing-tree-605c/K1.indfile)    [K = 1 Clumpp popfile](http://taylor0.biology.ucla.edu/structureHarvester/completedJobs/billowing-tree-605c/K1.popfile)

[K = 2 Clumpp indfile](http://taylor0.biology.ucla.edu/structureHarvester/completedJobs/billowing-tree-605c/K2.indfile)    [K = 2 Clumpp popfile](http://taylor0.biology.ucla.edu/structureHarvester/completedJobs/billowing-tree-605c/K2.popfile)

[K = 3 Clumpp indfile](http://taylor0.biology.ucla.edu/structureHarvester/completedJobs/billowing-tree-605c/K3.indfile)    [K = 3 Clumpp popfile](http://taylor0.biology.ucla.edu/structureHarvester/completedJobs/billowing-tree-605c/K3.popfile)

[K = 4 Clumpp indfile](http://taylor0.biology.ucla.edu/structureHarvester/completedJobs/billowing-tree-605c/K4.indfile)    [K = 4 Clumpp popfile](http://taylor0.biology.ucla.edu/structureHarvester/completedJobs/billowing-tree-605c/K4.popfile)

[K = 5 Clumpp indfile](http://taylor0.biology.ucla.edu/structureHarvester/completedJobs/billowing-tree-605c/K5.indfile)    [K = 5 Clumpp popfile](http://taylor0.biology.ucla.edu/structureHarvester/completedJobs/billowing-tree-605c/K5.popfile)

[K = 6 Clumpp indfile](http://taylor0.biology.ucla.edu/structureHarvester/completedJobs/billowing-tree-605c/K6.indfile)    [K = 6 Clumpp popfile](http://taylor0.biology.ucla.edu/structureHarvester/completedJobs/billowing-tree-605c/K6.popfile)

[K = 7 Clumpp indfile](http://taylor0.biology.ucla.edu/structureHarvester/completedJobs/billowing-tree-605c/K7.indfile)    [K = 7 Clumpp popfile](http://taylor0.biology.ucla.edu/structureHarvester/completedJobs/billowing-tree-605c/K7.popfile)

[K = 8 Clumpp indfile](http://taylor0.biology.ucla.edu/structureHarvester/completedJobs/billowing-tree-605c/K8.indfile)    [K = 8 Clumpp popfile](http://taylor0.biology.ucla.edu/structureHarvester/completedJobs/billowing-tree-605c/K8.popfile)

[K = 9 Clumpp indfile](http://taylor0.biology.ucla.edu/structureHarvester/completedJobs/billowing-tree-605c/K9.indfile)    [K = 9 Clumpp popfile](http://taylor0.biology.ucla.edu/structureHarvester/completedJobs/billowing-tree-605c/K9.popfile)

[K = 10 Clumpp indfile](http://taylor0.biology.ucla.edu/structureHarvester/completedJobs/billowing-tree-605c/K10.indfile)    [K = 10 Clumpp popfile](http://taylor0.biology.ucla.edu/structureHarvester/completedJobs/billowing-tree-605c/K10.popfile)

**Evanno method**

[*Evanno et al., 2005. Molecular Ecology 14, 2611 - 2620.](http://onlinelibrary.wiley.com/doi/10.1111/j.1365-294X.2005.02553.x/abstract) How are we calculating this? Look at the [FAQ.](http://taylor0.biology.ucla.edu/structureHarvester/faq.html)


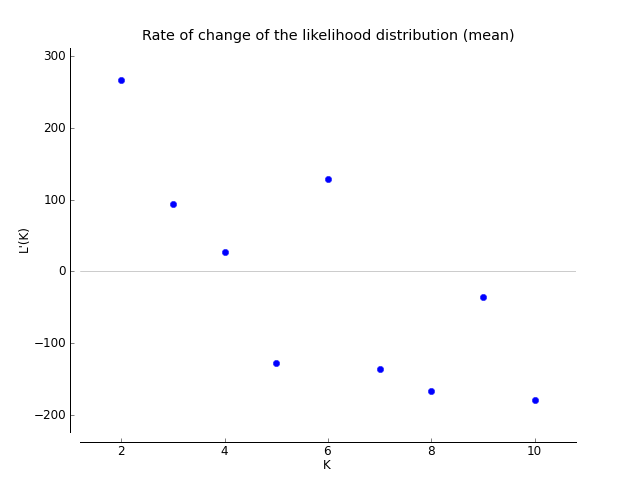


L'(K): [pdf](http://taylor0.biology.ucla.edu/structureHarvester/completedJobs/billowing-tree-605c/lnPK.pdf)   [eps](http://taylor0.biology.ucla.edu/structureHarvester/completedJobs/billowing-tree-605c/lnPK.eps)


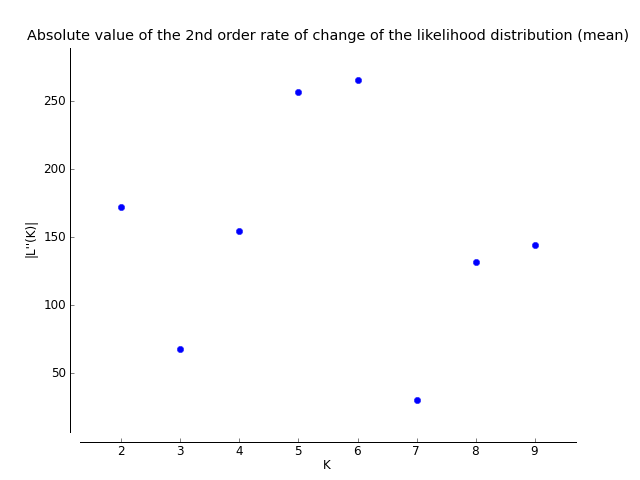


|L''(K)|: [pdf](http://taylor0.biology.ucla.edu/structureHarvester/completedJobs/billowing-tree-605c/lnPPK.pdf)   [eps](http://taylor0.biology.ucla.edu/structureHarvester/completedJobs/billowing-tree-605c/lnPPK.eps)


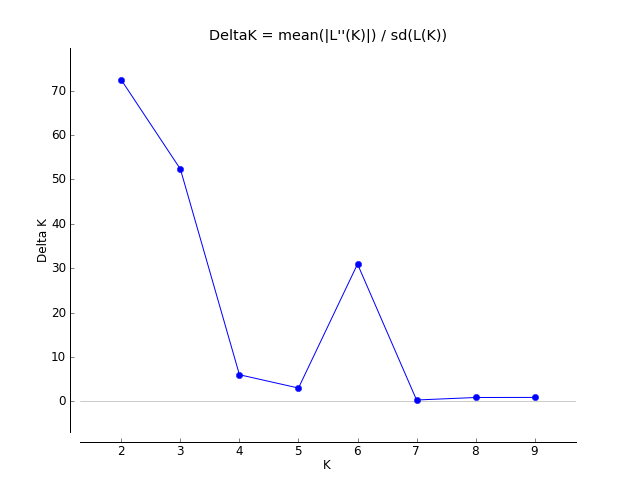


Delta K: [pdf](http://taylor0.biology.ucla.edu/structureHarvester/completedJobs/billowing-tree-605c/deltaK.pdf)   [eps](http://taylor0.biology.ucla.edu/structureHarvester/completedJobs/billowing-tree-605c/deltaK.eps)

The Evanno table output is also available as a tab-delimited text file (for use with Excel) [here](http://taylor0.biology.ucla.edu/structureHarvester/completedJobs/billowing-tree-605c/evannoTable.tab).

| **K** | **Reps** | **Mean LnP(K)** | **Stdev LnP(K)** | **Ln'(K)** | **\|Ln''(K)\|** | **Delta K** |
| --- | --- | --- | --- | --- | --- | --- |
| 1 | 10 | -4541.760000 | 0.374759 | — | — | — |
| 2 | 10 | -4275.130000 | 2.378165 | 266.630000 | 172.190000 | 72.404577 |
| 3 | 10 | -4180.690000 | 1.292242 | 94.440000 | 67.630000 | 52.335406 |
| 4 | 10 | -4153.880000 | 25.659947 | 26.810000 | 154.210000 | 6.009755 |
| 5 | 10 | -4281.280000 | 85.135380 | -127.400000 | 256.380000 | 3.011439 |
| 6 | 10 | -4152.300000 | 8.575935 | 128.980000 | 265.340000 | 30.940065 |
| 7 | 10 | -4288.660000 | 95.747401 | -136.360000 | 30.310000 | 0.316562 |
| 8 | 10 | -4455.330000 | 149.375731 | -166.670000 | 131.520000 | 0.880464 |
| 9 | 10 | -4490.480000 | 160.259497 | -35.150000 | 144.200000 | 0.899791 |
| 10 | 10 | -4669.830000 | 325.799605 | -179.350000 | — | — |
